# Comparison of oocyte vitrification using a semi-automated or a manual closed system in human siblings: survival and transcriptomic analyses

**DOI:** 10.1186/s13048-022-01064-3

**Published:** 2022-12-05

**Authors:** Julie Barberet, Bastien Ducreux, Céline Bruno, Magali Guilleman, Raymond Simonot, Nicolas Lieury, Adrien Guilloteau, Déborah Bourc’his, Patricia Fauque

**Affiliations:** 1 Université Bourgogne Franche-Comté - Equipe Génétique des Anomalies du Développement (GAD) INSERM UMR1231, 2 Rue Angélique Ducoudray, F-21000 Dijon, France; 2 CHU Dijon Bourgogne, Laboratoire de Biologie de la Reproduction – CECOS, 14 rue Gaffarel, F-21000 Dijon, France; 3 USMR, Dijon Bourgogne University Hospital, F-21000 Dijon, France; 4 Institut Curie, PSL University, CNRS, INSERM, 26 rue d’Ulm, F-75248 Paris, France; 5 Laboratoire de Biologie de la Reproduction, CHU Dijon, BP 77908, 14, rue Gaffarel, 21079 Dijon Cedex, France

**Keywords:** Assisted reproductive technology, Cryopreservation, Oocyte, Single-cell RNA-seq, Vitrification

## Abstract

**Background:**

Indications of oocyte vitrification increased substantially over the last decades for clinical and ethical reasons. A semi-automated vitrification system was recently developed making each act of vitrification reproducible. In this study, we evaluated the efficiency of the semi-automated technique of oocyte vitrification by survival rate, morphometric assessment and resistance to empty micro-injection gesture as compared with a manual method. Additionally, we intended to evaluate transcriptomic consequences of both techniques using single-cell RNA-seq technology.

**Results:**

Post-warming survival rate, oocyte surfaces and resistance to empty micro-injection were comparable between semi-automated and manual vitrification groups. Both oocyte vitrification techniques showed limited differences in the resulting transcriptomic profile of sibling oocytes since only 5 differentially expressed genes were identified. Additionally, there was no difference in median transcript integrity number or percentage of mitochondrial DNA between the two groups. However, a total of 108 genes were differentially expressed between fresh and vitrified oocytes (FDR < 0.05) and showed over-represented of genes related to important cellular process.

**Conclusions:**

Our results provide reassurance about the influence of semi-automation as compared with the manual vitrification method. Concerning oocyte vitrification itself, no tight common transcriptomic signature associated has been observed across studies.

**Trial registration:**

NCT03570073.

**Supplementary Information:**

The online version contains supplementary material available at 10.1186/s13048-022-01064-3.

## Background

Following its introduction, and despite the first birth from a frozen oocyte in 1986 [10], oocyte cryopreservation remained a challenging technique for many years. The unique structure of mature oocytes, with a low surface area to volume ratio, makes them particularly sensitive to osmotic stress and high concentrations of cryoprotectant. As an alternative to slow freezing, the introduction of vitrification led to improved outcomes. Authorized in the United Kingdom since 2000 and classified as a non-experimental technique by the American Society for Reproductive Medicine (ASRM) in 2013, survival rates after vitrification have surpassed slow freezing [35], becoming the norm in terms of oocyte cryopreservation. Consequently, indications and the number of oocyte vitrifications are rising worldwide in the field of assisted reproductive technology (ART). In addition to fertility preservation in cases of infertility risk due to gonadotoxic treatments or other medical conditions, oocyte vitrification is used for egg banking in donation programs, to postpone embryo transfer to prevent ovarian hyperstimulation, to build larger cohorts for poor responders, or to delay childbearing [11].

While early randomized controlled trials reported reproductive outcomes similar to those achieved with fresh oocytes [12, 31, 36], some recent cohort studies highlighted lower reproductive outcomes with the use of vitrified oocytes [13, 14, 25]. The loss of oocytes after thawing, leading to a diminished pool of oocytes available for insemination, is likely a reason for the inferior clinical results reported in these studies [13]. Thus, variations in the efficiency of the vitrification/warming technique performed may lead to lower reproductive outcomes. The event of degeneration after warming is known to be centre- and operator-dependent [21]. Qualified as a highly skilled procedure requiring precision and speed, vitrification thus needs high-trained operators. To improve reproducibility and consistency of processes, a semi-automated system was developed and is commercially available for clinical use: Gavi® (Genea, Sydney, Australia). Important variables such as temperature, exposures times, media volume and replacement are entirely automated, suggesting that the survival rate and inter-operator variability could be improved. At the same time, making each act of vitrification is traceable. Assessment of the efficiency of the semi-automated platform as compared with the reference method (manual) was recently made at the zygote [22], cleaved embryo [20] or blastocyst stages [29]. However, there is no comparative study on the expected oocyte survival rate using the Gavi® system.

In addition to concerns about efficiency of oocyte vitrification, there are safety apprehensions in terms of the vulnerability of intracellular content and structures. To date, much of our knowledge is obtained from animal models, but there are inherent differences between oocyte maturation and fertilization in animals and humans, which limits the generalization of findings relative to molecular alterations following oocyte vitrification to human [51]. So far, six studies have investigated the impact of oocyte cryopreservation on gene expression in humans, but none of them compared the vitrification method (i.e. manual vs semi-automated) [9, 15, 16, 23, 30, 41]. Among them, only one used single-cell RNA-sequencing technology (scRNA-seq) on 16 oocytes [23]. In this study, the transcriptome of the vitrified oocytes was altered notably through degradation of RNA integrity and the down-regulation of genes involved in major cell cycle and development processes, but there was no control group from the same batch experiment [23].

It is therefore of utmost importance to comprehensively assess the efficiency and transcriptional “safety” of oocyte vitrification as well as the recent semi-automated technique of vitrification provided by the Gavi® system as compared with the manual method. The primary objective of the current study was thus to investigate survival using of both methods of vitrification with a sibling oocyte design. Secondly, we investigated the transcriptomic landscape associated with the oocyte vitrification method using scRNA-seq. Finally, we compared the scRNA-seq data obtained after vitrification with those from a cohort of fresh sibling oocytes.

## Results

**Fig. 1:**
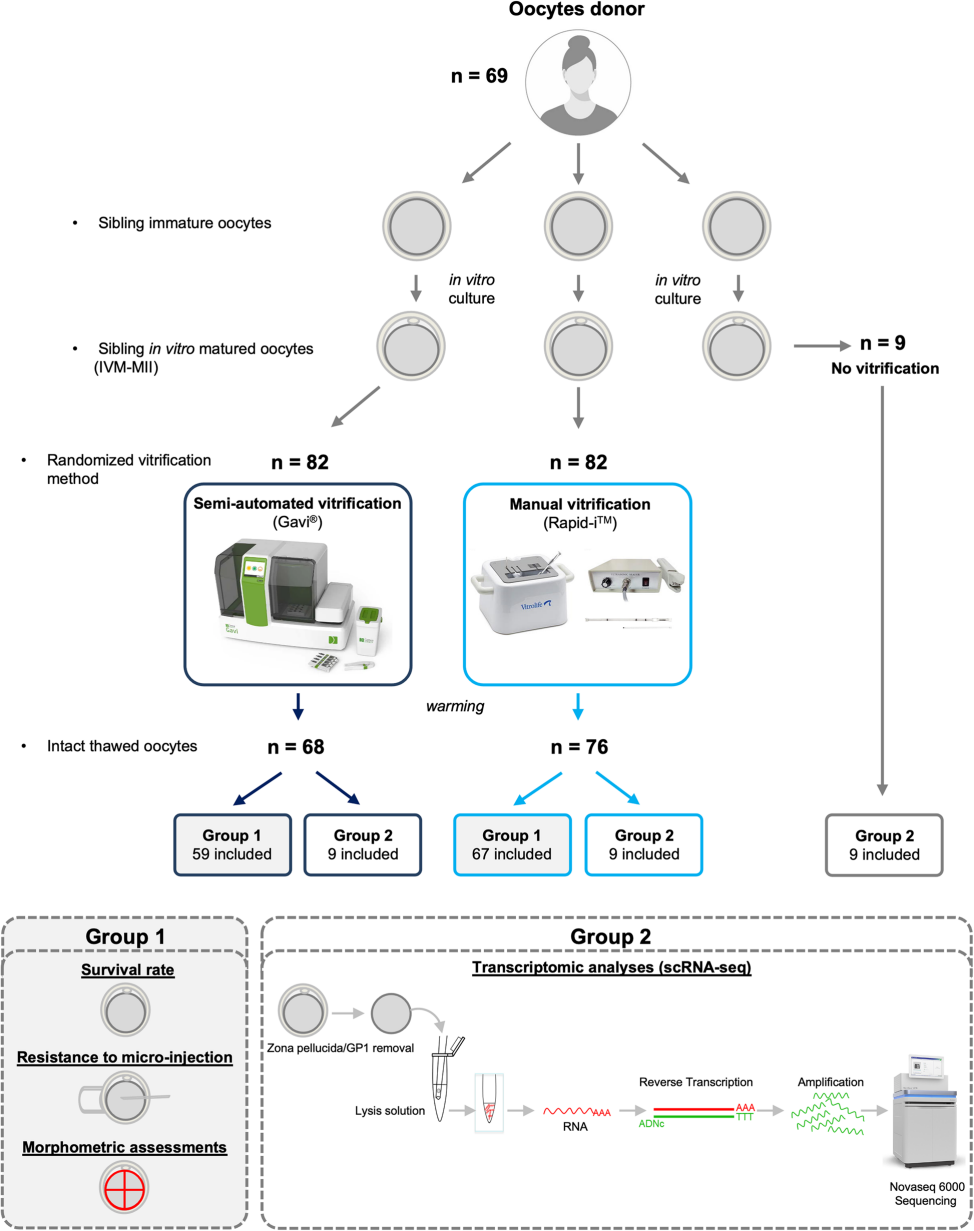
Summary of the study design. After patient selection and oocyte collection, sibling oocytes were allocated to two different groups; Group 1: Manually or semi-automatically vitrified sibling oocytes for morphometric, resistance to micro-injection and survival rate assessments. Group 2: Fresh, manually or semi-automatically vitrified sibling oocytes for transcriptomic analyses by scRNA-seq

Oocytes were prospectively collected from 69 donors of sibling *in vitro* matured oocytes, aged 33.3 ± 4.7 years old. In total, 173 sibling oocytes were randomly allocated between three groups: 82 in the semi-automated and manual vitrification, and 9 in the “fresh” group. The Fig. 1 summarizes the inclusion flow-chart.

### Survival rate, resistance to micro-injection, morphometric assessment

A total of 164 oocytes were thawed (Table I). The post-warming survival rate was comparable between the two groups, despite the difference being near the statistical threshold for significance: 82.9% (68/82) and 92.7% (76/82) in the Gavi® and Rapid-I™ groups, respectively (OR: 2.91, 95% CI: 0.98–8.63, *p* = 0.053). Among the intact oocytes included in Group 1 and subjected to an empty micro-injection gesture three hours after warming, the survival rate was comparable between the two groups: 93.2% (55/59) and 94.0% (63/67) in the Gavi® and Rapid-ITM groups, respectively (OR: 1.16, 95% CI: 0.27–5.03, *p* = 0.837).

**Table I:** Survival rate after thawing and resistance rate after micro-injection gesture

	Manual	GAVI	OR (95%CI)	***p-value***^***a***^
No. warmed oocytes	82	82		
No. survived oocytes	76	68		
Survival rate	92.7%	82.9%	2.91 (0.98–8.63)	0.053
No. injected oocytes	67	59		
No. viable oocytes post injection	63	55		
Post injection viability	94.0%	93.2%	1.16 (0.27–5.03)	0.837

For categorical variables, n (%) is presented. *OR* Odds Ratio; *95%CI* 95% Confidence Interval

^a^Logistic mixed models with random effect on donor were estimated

**Fig. 2:**
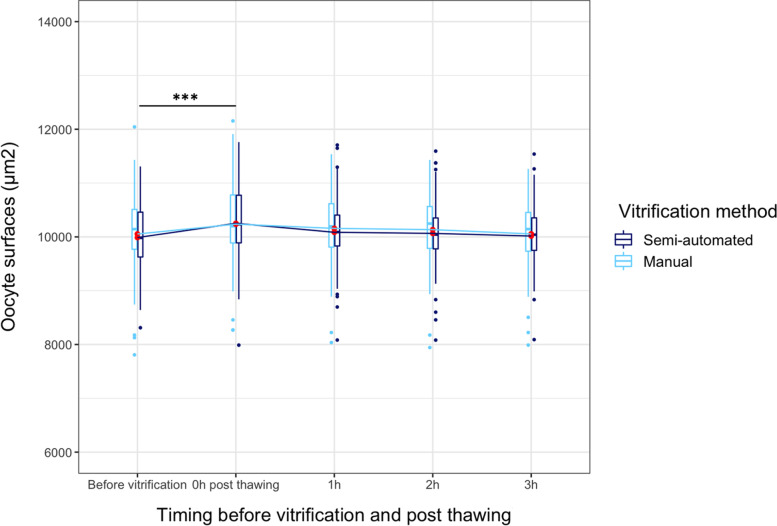
Oocyte surface according to the timing before vitrification and post thawing. All comparisons were not significant except between oocyte surfaces before and immediately post-thawing. Significance was assessed with linear mixed model with random effects. ***: *p* < 0.001

Reflecting the post-thawing rehydration of the oocytes, oocyte surfaces were comparable between the semi-automated and the manual group (Additional file 1) and followed the same rehydration kinetics when surface before vitrification is used as a reference (Fig. 2). Immediately after thawing, the oocyte surface was reduced, whatever the mode of vitrification, compared with the same oocytes before vitrification (*p* < 0.001). However, the difference did not persist one hour after post-thawing (*p* = 0.068).

### Single-cell RNA-seq assessment

Nine oocyte donors were included, and single-cell RNA sequencing (scRNA-seq) analysis was conducted on 20 oocytes from 7 oocytes donors for technical reasons. Among them, six patients donated *in vitro* matured oocytes (IVM-MII) and one donor was part of an oocyte donation program and donated 3 MII oocytes.

**Fig. 3:**
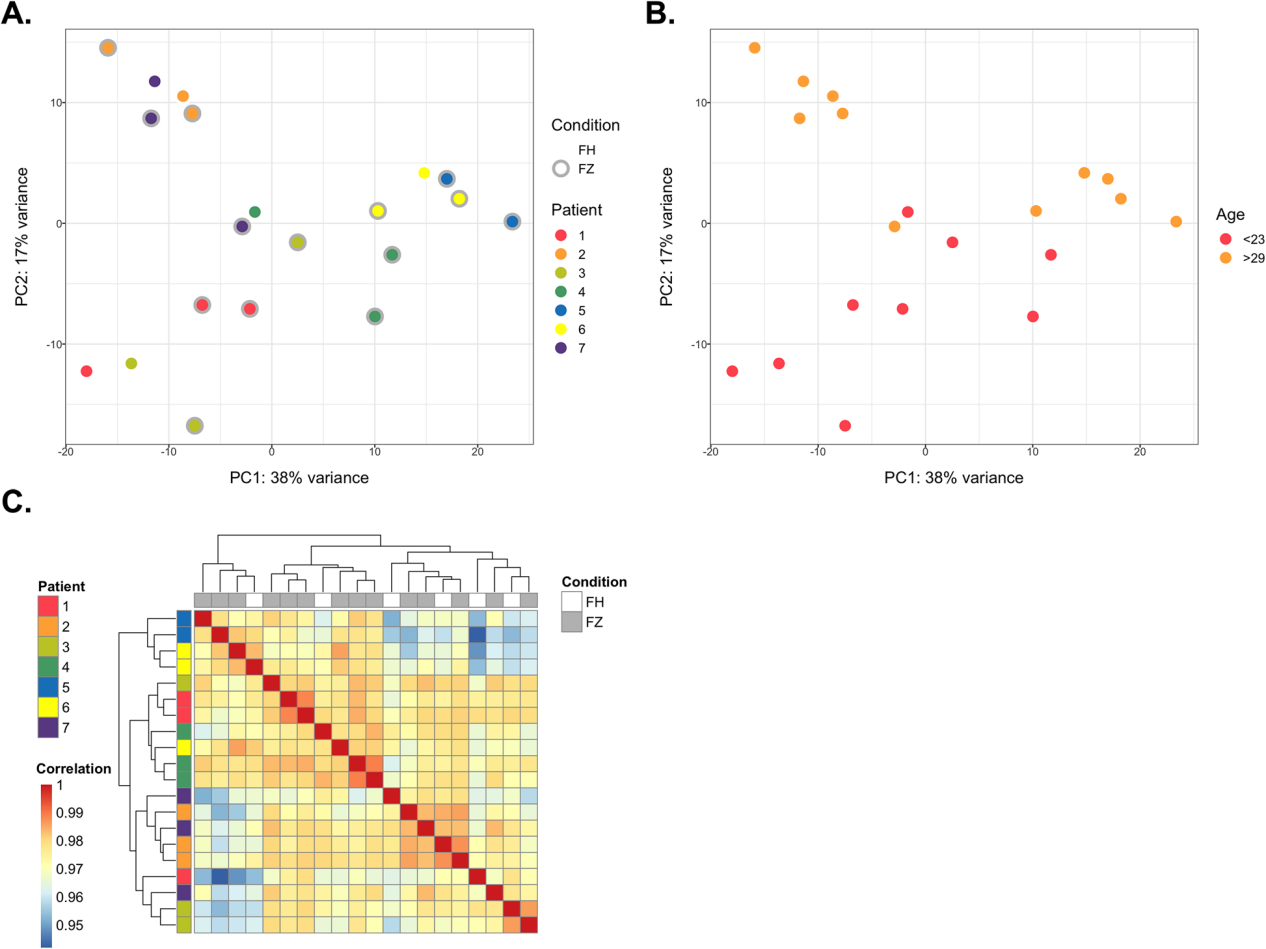
Global transcriptome analysis of oocytes according to sample characteristics. **A** Principal component analysis of all samples highlighting the vitrification status and the patient origin. PC: principal component. FH: fresh oocyte. FZ: frozen oocyte. **B** Principal component analysis of all samples highlighting the patients’ age. **C** Unsupervised hierarchical clustering of samples according to their global gene expression

After quality checks and filters, 12,808 transcripts expressed across all samples were subjected to analysis, which is consistent with previous studies [18, 46]. The medTIN of the samples ranged between 32.3 and 46.0, and reads were uniformly distributed along the gene body, reflecting the high quality of our samples (Additional file 2). To identify potential confounders in our dataset, we performed a principal component analysis (PCA) to visually inspect the similarity between samples according to their log-transformed gene counts (Fig. 3A and B). The first two principal components (PC1 and PC2) separated samples according to patient effects such as maternal age rather than whether oocytes were vitrified or not. This was further demonstrated by unsupervised clustering of log transformed gene counts in which samples nearly clustered together according to their patient’s origin (Fig. 3C).

#### Manual vs. semi-automated vitrification

**Fig. 4:**
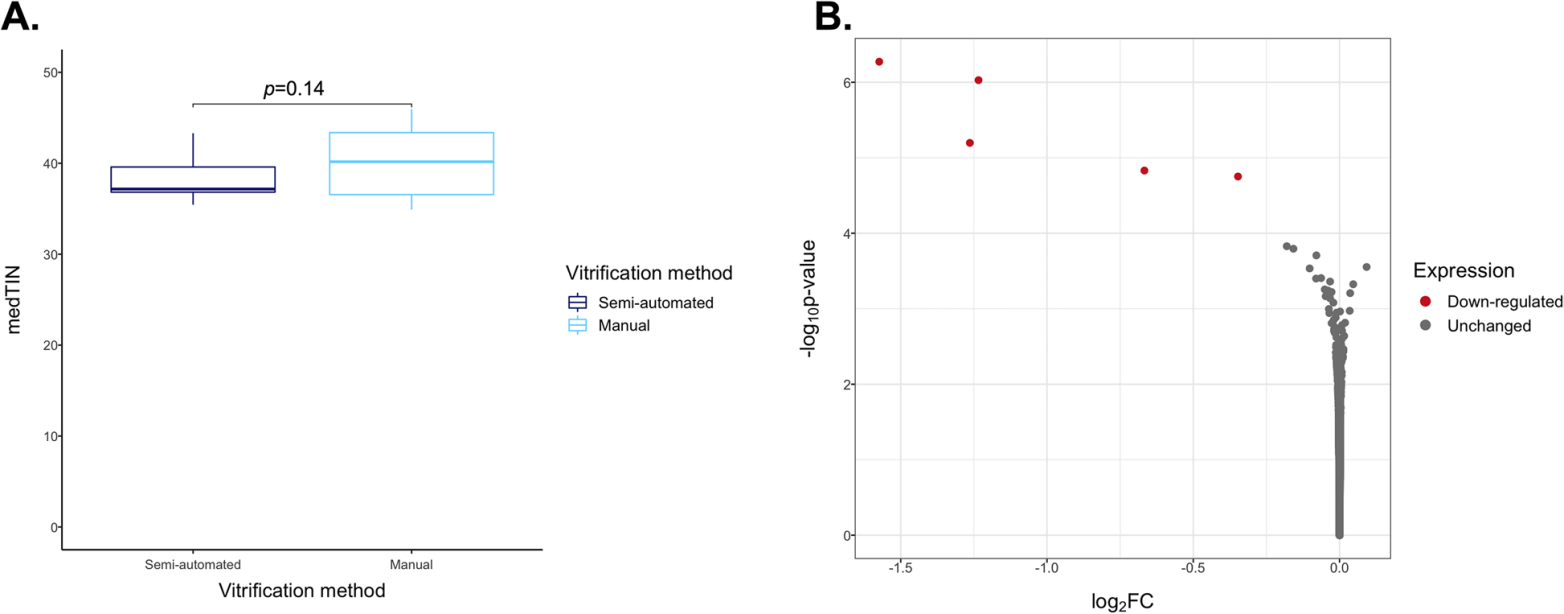
Differential gene expression and RNA quality of semi-automatically and manually vitrified oocytes. **A** Comparison of mean medTIN between semi-automated and manual vitrification groups. Significance was assessed using a linear mixed model with random effects. **B** Volcano plot representation of the differential expression analysis with semi-automated vitrification compared with manual vitrification (Semi-automated minus Manual). Each point represents one of the genes expressed with log2(FC) on the x-axis and –log10 of the unadjusted *p*-value calculated with DESeq2 on the y-axis

Firstly, to elucidate whether the type of vitrification process had an impact on the oocyte transcriptome, scRNA-seq libraries were generated from seven pairs of sibling oocytes following semi-automated or manual vitrification. RNA integrity was similar between the two modes of vitrification because the medTIN was not different between the two groups (Fig. 4A, *p* = 0.14). Oocytes exhibited limited differential expression between these sample groups since only 5 DEGs were identified: *ARSD*, *CCDC124*, *CLPS*, *PLCH2*, *RHBDF1*. All were upregulated with semi-automated vitrification and 3 of them showed absolute log2(FC) > 1: *CCDC124*, *CLPS*, *PLCH2*, (Fig. 4B). These five genes have a low level of expression in oocytes (Additional file 3), and no interaction between them has been recorded in the STRING database. Comparing the expression profile of *in vivo* mature oocytes, no genes were differentially expressed depending on the mode of vitrification, revealing limited effects (Additional file 4).

#### Fresh vs. vitrified oocytes

**Fig. 5:**
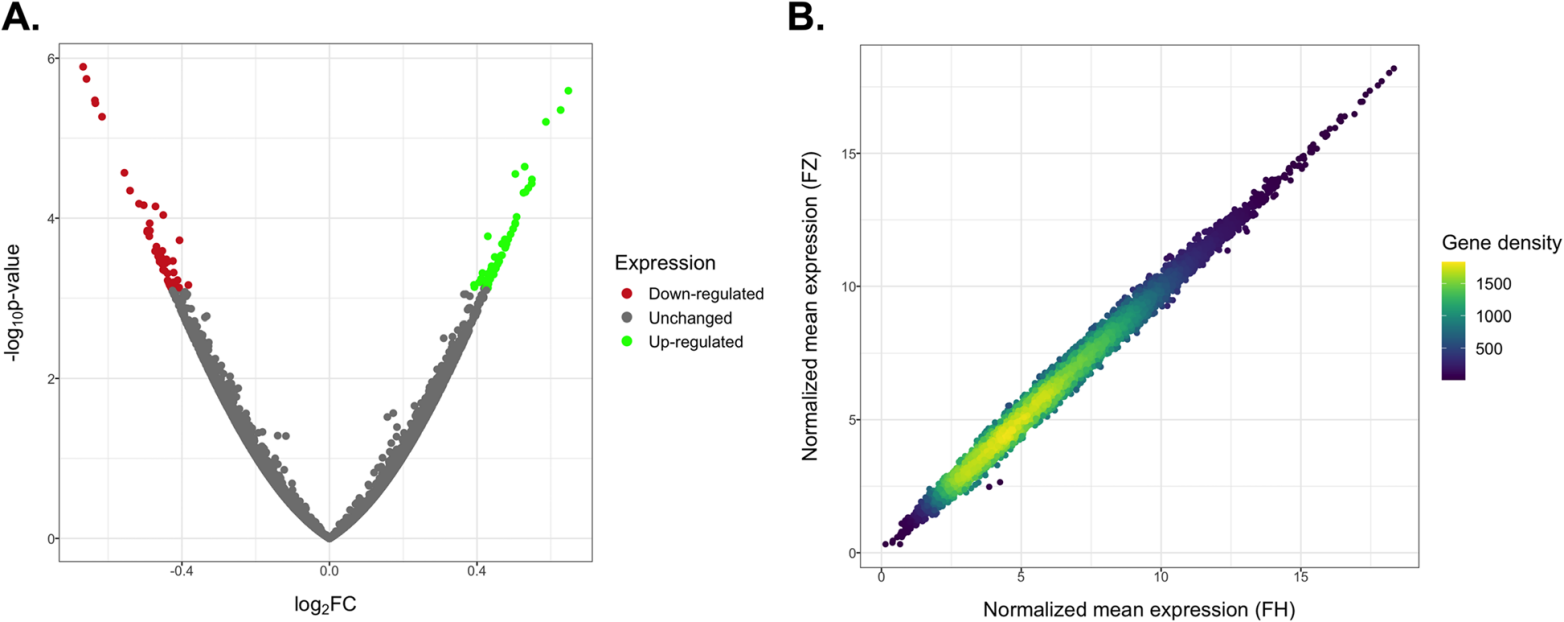
Differential gene expression analysis of FH and FZ oocytes. **A** Volcano plot representation of the differential expression analysis with FZ compared with FH oocytes (FZ minus FH). **B** Correlation of the normalized mean expression (relative log expression computed with DESeq2) between FH and FZ oocytes for each gene

**Fig. 6:**
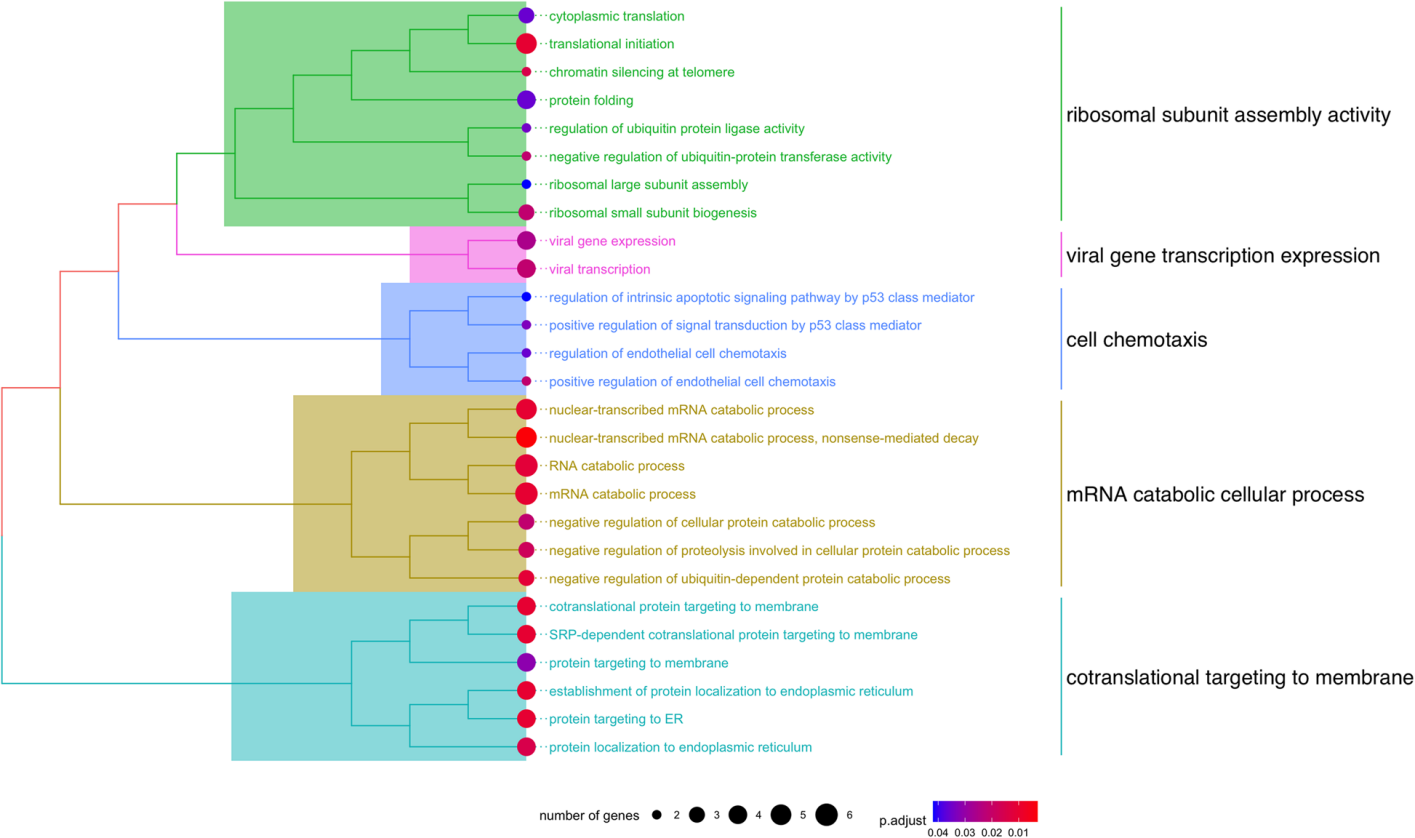
Hierarchical clustering of the significant ontologies related to the down-regulated DEGs with vitrification. Each group of ontologies was assigned a denomination according the semantic similarity of the enriched biological processes

Secondly, our study design allowed us to test for the potential effects of vitrification in the transcriptome of MII oocytes, whatever the mode of vitrification. We therefore performed a differential expression analysis between fresh (FH) versus vitrified (FZ) oocytes while accounting for individual baseline expression for each patient. We observed no degradation of the RNA quality in vitrified oocytes (*p* = 0.02, but indicating vitrified oocytes had higher TIN) and vitrification had no effect on mitochondrial proportion (*p* = 0.41) (Additional file 2). A total of 108 differentially expressed genes (DEGs) (54 up-regulated, 54 down-regulated) emerged, but none of them reached log2(FC) > 1, revealing small significant differences between the two conditions (Fig. 5A, Additional file 5). The high comparability of the transcriptome in FH vs FZ oocytes can be detected by comparing the mean expression level of FH and FZ oocytes (Pearson’s correlation, Coefficient = 0.996) (Fig. 5B). Seven of our DEGs were common with the study of Huo et al. [23], but all displayed the opposite variation of expression with vitrification (*DYSF*, *KIAA0319L*, *USP4*, *TRPC3*, *SH3RF1*, *FOXO3B*, *ZNF530*). We further explored the biological relevance of the 108 DEGs by subjecting them to Gene Ontology over-representation analysis. Two pathways reached statistical significance (FDR < 0.05) and referred to mRNA catabolic process and ribonucleoprotein complex biogenesis. An over-representation analysis on subsets of DEGs revealed no significant pathways for up-regulated DEGs but 27 significant pathways for down-regulated DEGs, mainly related to mRNA and RNA catabolic processes (Fig. 6).

**Fig. 7:**
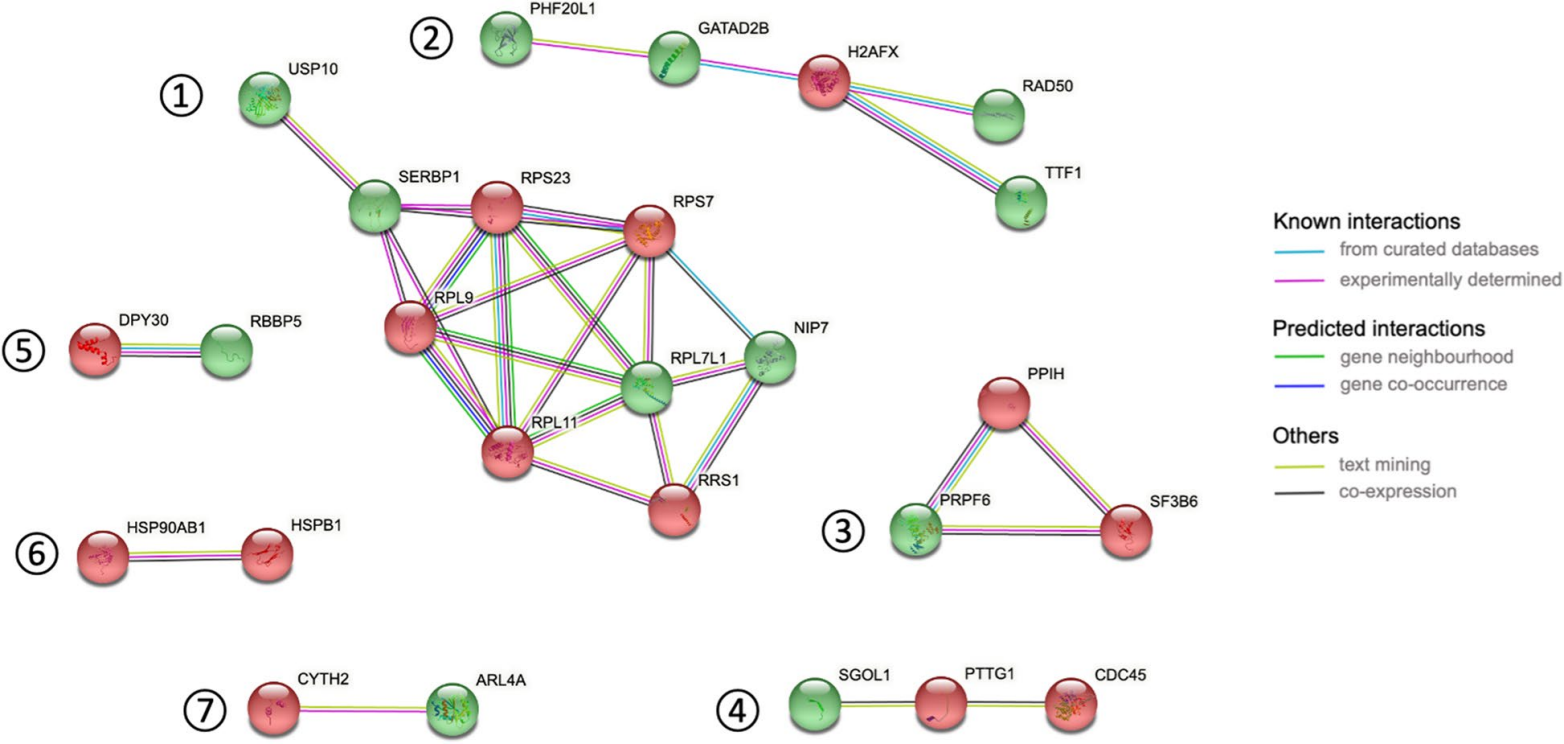
Protein-protein interaction network of DEGs between FH and FZ oocytes. Genes highlighted in green and red are respectively up- and down-regulated with vitrification

The protein-protein interaction analysis of DEGs in Fig. 7 displays high-confidence networks. A single network involved more than 5 proteins and consisted in interactions of proteins related to ubitiquin hydrolysis, RNA-binding and ribosomal biogenesis (network 1). Secondary networks were related to transcriptional regulation (network 2), pre-mRNA splicing (network 3), cell division control and chromosome stability (network 4), methylation of histone 3 (network 5), heat shock proteins involved in cell cycle control (network 6) and guanine nucleotide exchange (network 7).

**Fig. 8:**
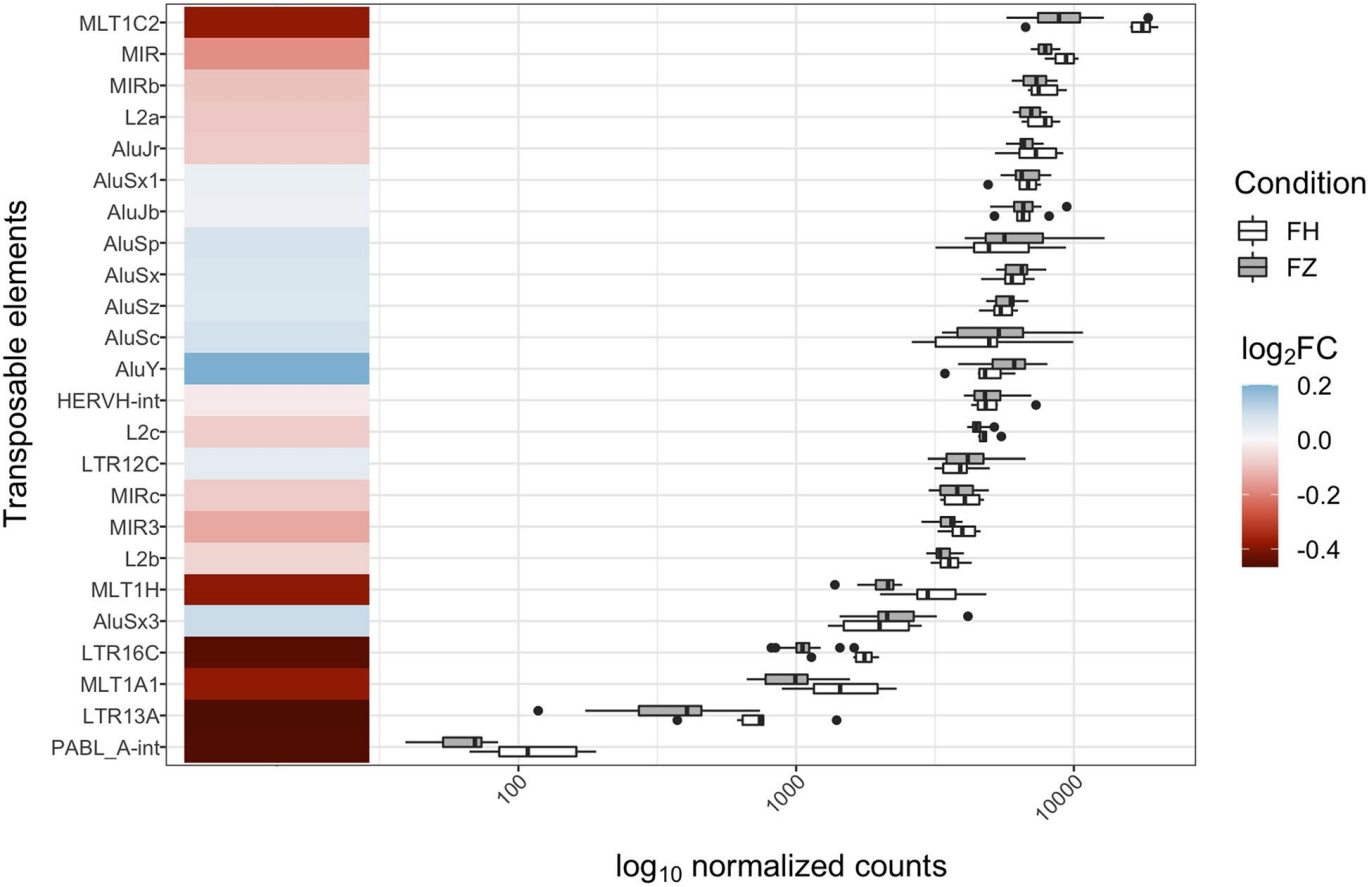
Overview of the expression level of the 20-most expressed transposable elements between FH and FZ oocytes and the four differentially expressed TE with vitrification, ranked by expression

Using an extensive genome annotation, we quantified the expression of transposable elements (TEs). Four TEs were present in the 108 DEGs, and all of the TEs were downregulated with vitrification (LTR16C, MLT1A1, LTR13A, PABL_A-int). These elements are not among the most expressed TEs in the oocyte genome (a heatmap of the top 20 most expressed TEs and their expression profile is shown in Fig. 8). We also specifically checked for the expression of DNA methylation-related genes (*TET2*, *TET3*, *TDG*, *DNMT1*, *UHRF1*, *DNMT3A*, *DNMT3B*), but we did not observe any difference between the vitrified and fresh oocytes groups (Additional file 6).

## Discussion

The introduction of Gavi®, a semi-automated vitrification device, provides an opportunity to improve the standardization of incubation duration, temperatures, and cooling rates in order to increase survival rates and lower inter-operator variability. To date, there is no available data for oocyte outcomes in terms of efficiency and safety.

The first aim of this unique study was to assess the efficiency of the semi-automated closed Gavi® oocyte vitrification method as compared with a manual closed Vitrolife method. Even if survival rates following manual vitrification tended to be higher, we reported statistically comparable survival rates between techniques, which was similar to what has been previously reported in the literature at different stages of embryonic development. Indeed, vitrification with the Gavi® system previously showed no difference in survival rate after vitrification at the zygote [22] or blastocyst stage [29] as compared with the manual open Cryotop® method. Comparable intact survival rate (with 100% intact blastomeres) and clinical pregnancy rate were recently reported after embryo vitrification at Day 2 or 3 after Gavi® vitrification than manually with Irvine®-CBS® system [20]. In the current study, we also investigated the degree and kinetics of post-thawing rehydration through morphometric measurements, as well as thawed-oocyte resistance to a micro-injection gesture without spermatozoa. There was no difference in morphometric assessments and in the proportions of oocyte lysis after micro-injection between the vitrification methods. The results for the viability of oocytes provided by the semi-automated system compared with the closed manual method thus seem reassuring, suggesting that it could be used as an alternative vitrification procedure in ART.

The second aim of this study was to assess the impact of the semi-automated method on the transcriptome and to enrich current knowledge about the transcriptomic effects of vitrification, which has not yet been thoroughly explored in the literature. We performed single-cell RNA-sequencing with a high throughput method. The transcriptomic profiles obtained with semi-automated and manual vitrification revealed similar patterns except for three DEGs with absolute log2(FC) > 1. Among them is *CLPS*, which is a colipase involved in lipid hydrolysis whose downregulation has already been observed in relation to oocyte aging [7]. The two remaining DEGs encode for a phospholipase (*PLCH2*), and a coiled-coil domain containing protein required for proper progression of late cytokinetic stages (*CCDC124*). In addition, there was no degradation of the RNA integrity caused by any of the vitrification method in our samples according to the medTIN measure. This result is reassuring as to the preserved quality of the transcriptional material stored in the oocyte, which will be necessary for the proper development of the future embryo.

Finally, our study design made it possible to comprehensively compare the transcriptome of fresh and vitrified oocytes originating from the same patient. Among the differentially expressed genes, the overall magnitude of the differences remains largely limited between the two cohorts of sibling oocytes, reinforcing the reports of Di Pietro et al. [16] and D’Aurora et al. [15], based on a targeted approach using RTqPCR. However, the transcriptional differences between fresh and vitrified oocytes were reported more extensive by two other studies using genome-wide approach [23, 30]. Huo et al. [23], with scRNA-sequencing technology, and Monzo et al. [30], with pooled microarray, respectively found 1987 and 608 DEGs associated with vitrification, with a decrease in the mRNA content for the large majority of the DEGs. A decrease in the mean expression of 18 genes tested with a PCR approach was also reported by Chamayou et al. [9]. No tight common transcriptomic signature associated with vitrification has been observed across studies, including ours. This is potentially the result of disparate techniques and questionable study designs. Unlike PCR and microarrays, scRNA-seq has the advantage of an unbiased vision of all transcripts. Additionally, the choice of an appropriate control group should be tightly controlled and for this reason we opted for a paired design consisting in triplets of oocytes from the same patients. Indeed, one limitation of the study by Huo et al. [23] stems from the choice of the fresh oocytes comparison group, which came from a previously published experiment [46] and may have led to difficulties distinguishing between the batch and vitrification effects. However, a consensus towards the disturbance of the RNA process, cell cycle process, meiotic process, response to stress and ubiquitination may occur following vitrification. Interestingly, the down-regulation of genes involved in important cellular processes has been a recurrent observation in studies on transcriptomic effects in relation to vitrification. In our study, up-regulated genes were likely to function independently seeing as they were not related to common genetic ontologies, contrary to down-regulated genes. However, applying the same statistical threshold set in Huo’s and Monzo’s genome-wide studies (absolute log2(Fold Change) > 1), no significant genes (FDR < 0.05) were detected in the current study. In addition, there was no change in RNA integrity, as highlighted by the comparable medTIN between our two groups. This result was not in accordance with Huo et al. [23], who observed a drastic decrease in RNA integrity associated with vitrification [23], but the TIN is highly variable depending on sample processing.

As our team recently pointed out, the control of transposable elements is crucial for gametes and embryos [5]. In mouse oocytes, over-expression of LINE-1 elements was associated with aneuploidy and embryonic death [28]. The mechanisms regulating TEs involve epigenetic events driven by microRNAs, histone modifications and DNA methylation [8, 38, 44]. Despite being a major concern, epigenetic changes in TEs following human oocyte vitrification have not yet been assessed with high-throughput sequencing. For the first time, we tested the impact of vitrification on repetitive elements with an extensive annotation database of TE families, particularly the long terminal repeat (LTR) families, which are highly expressed in the oocytes and regulate the expression of host genes in the early embryo [32]. Four elements which are endogenous retroviruses (which belong in the LTR retrotransposons class) were significantly downregulated in the vitrified oocytes group, but they are not among the most expressed families in human oocytes and the differences were very limited. Vitrification has also been suggested to reduce the copy number of mitochondrial DNA in mice and cows [2, 4]. mtDNA is a key determinant in oocyte quality and low levels of mtDNA might indicate low developmental competence [34, 39]. Reassuringly, we observed no difference in mitochondrial proportion between the fresh and frozen oocyte groups.

To date, this is the only study to investigate the oocyte survival rate with semi-automated vitrification process and the biggest scRNA-seq experiment on vitrified oocytes performed. However, most of our oocytes underwent an *in vitro* maturation step. Even though the *in vitro* maturation of the oocyte could be associated with some changes in the gene expression profiles assessed by scRNA-seq [48, 50], the oocyte sibling design allowed us to draw strong conclusions when we compared the two techniques. In addition, we confirmed that there was no difference between the two vitrification methods for donor oocytes that were matured *in vivo*. One of the key strengths of this study is that it was able to compare oocytes from the same patient with the use of sibling oocytes. This study design allowed us to control for potential confounders related to patient effects, so the survival rates and transcriptomic profiles were not related to the patient’s characteristics.

This study is only the second to analyse the differential expression of genes by sc-RNAseq in a single oocyte rather than in a pool of oocytes. Further validation, for instance with recently developed single-cell multi-omics, would be valuable for comprehensively understand the relationship with transcriptional modifications observed after vitrification and DNA methylation dynamics, chromatin accessibility and histone modifications [47].

## Conclusions

This study provides the first results on oocyte vitrification efficiency and the transcriptional impact of the automated Gavi® system compared with a closed manual vitrification method. Based on a sibling oocyte design, we showed that the oocyte survival rate, morphometric measures, and resistance to micro-injection are similar in the two methods.

In addition, for the first time, we explored the transcriptomic profiles of both techniques using single-cell RNA-sequencing. The fact that the observed transcriptional changes were infrequent and of low intensity provides reassurance about the influence of semi-automation when compared with the manual method. Our study highlights the specific adverse effects of oocyte vitrification on genomic expression. Research in this field must be continued considering that fertility preservation through oocyte vitrification is bound to develop worldwide in the coming years. Comparing clinical outcomes such as fertilization, cleavage, embryo quality, pregnancy rates, implantation, births as well as monitoring the health of children following oocyte vitrification via both techniques would also be needed in the future.

## Methods

### Patient selection and oocyte collection

This study was prospectively conducted at the University Hospital of Dijon between June 2018 and February 2020. Patients undergoing an attempt of *in vitro* fertilization with microinjection (ICSI, IntraCytoplasmic Sperm Injection) for medical reasons and patients who were part of an oocyte donation program were informed of this study, and their written consent was obtained before oocyte stimulation. Oocyte collection was declared (Clinical Trial NCT03570073) and approved by the ethics committee (2017-A02444–49). The controlled ovarian hyperstimulation and oocyte retrieval were performed as previously described [6].

On the day of oocyte retrieval, after removing the cumulus cells (hyaluronidase treatment plus mechanical pipetting), oocytes were classified as mature (Metaphase II) or immature (Germinal Vesicle or Metaphase I oocyte). When more than one immature oocyte was present, they were cultured in Global culture media (Global, LifeGlobal, USA) in a time-lapse incubator (EmbryoScope, Unisense FertiliTech, Denmark), at 37.0 °C, 6%CO2, 5%O2 for a maximum period of 24 hours. Images and related data were stored in the EmbryoViewer (Unisense FertiliTech, Denmark) and subsequently analyzed. Oocytes that had progressed to the MII stage within the first 24 h of culture (IVM-MII stage) were selected for subsequent inclusion. Patient inclusion was based on the presence of more than two IVM-MII (siblings).

With two or an even number of IVM-MII, sibling oocytes were included in Group 1 “Survival rate, morphometric assessment, resistance to micro-injection”. In that case, IVM-MII siblings were randomly and equally assigned into manual or semi-automated vitrification groups.

When there were three IVM-MII, sibling oocytes were included in Group 2: “scRNA-seq assessment”. In this case, sibling oocytes were randomized between fresh, manual or semi-automated vitrified oocytes, and single-cell transcriptomes were analyzed and compared. Three *in vivo* MII oocytes were also included in Group 2 on the day of oocyte retrieval from a patient who was part of an oocyte donation program.

### Oocyte conditioning

Vitrification and warming processes were performed by the two same operators.

#### Manual vitrification-warming procedure (Reference method)

MII were vitrified in a closed system (Rapid-I™, Vitrolife, Sweden) using the RapidVit™ Oocyte kit (Vitrolife, Sweden) containing ethylene glycol and propanediol, which are permeable cryoprotectants, and sucrose, acting as an extracellular cryoprotectant. The vitrification procedure was performed according to the manufacturer’s protocol. Each solution (Vitri 1™ Oocyte, Vitri2™ Oocyte and Vitri 3™ Oocyte) was warmed to 37 °C in a multi-well plate. The oocyte was transferred for 7 minutes in Vitri 1™ Oocyte, 4 minutes in Vitri 2™ Oocyte, and a maximum of 25 seconds in drops of Vitri 3™ Oocyte including the loading on cryodevice (Rapid-I) and immersion into liquid nitrogen. The vitrified oocytes were warmed with a RapidWarm™ Oocyte kit (Vitrolife, Sweden) at 37 °C. The Rapid-I was plunged into Warm 1™ Oocyte for 1 minute. The oocyte was then moved into Warm 2™ Oocyte for 3 minutes, Warm 3™ Oocyte for 5 minutes, and Warm 4™ Oocyte for 7 minutes. Immediately after warming, oocytes were loaded into the Embryoscope with Global media (Global, LifeGlobal, Denmark) for morphometric assessments or in a 4-well culture dish for transcriptomic assessment.

#### Semi-automated vitrification and warming with the GAVI® system

MII oocytes were vitrified in the semi-automated vitrification group using GAVI®, as previously described by Roy et al. [37]. Oocytes were first equilibrated in VitBase solution for 5 minutes in an un-gassed incubator at 37 °C. In the meantime, the medium cartridge containing vitrification solutions VS1 and VS2 (ethylene glycol, dimethyl sulfoxide, trehalose and supplemented human serum albumin) and tip and seal cartridges were loaded into the GAVI® system. The oocytes were then individually loaded into a device called a “Pod” and loaded into GAVI®. The oocyte vitrification protocol was selected, run and once finished, the cassette was manually removed and dunked into liquid nitrogen. Oocytes were stored in a liquid nitrogen tank before being warmed as per Genea BIOMEDX protocol using Gems Warming solutions® (Genea, Sydney, Australia). After a brief warming in a 37 °C water batch, 10 μl of WarmSol1 was dispensed in less than 20 seconds in the Pod, over a period of 1 minute. Oocytes were then transferred for 1 minute in WarmSol1, 3 minutes in WarmSol2, 5 minutes in WarmSol3, 1 minute in WarmSol3 before being transferred into Global media (Global, LifeGlobal, Denmark).

### Survival rate, Morphometric assessment, Resistance to micro-injection

The primary outcome of this study was the survival rate (i.e. the number of intact oocytes after thawing divided by the total number of warmed oocytes). To evaluate post thawing oocyte rehydration for oocytes included in Group 1, the oocyte surfaces (i.e. ooplasm surface) were estimated with the Embryoscope tool immediately after thawing, and then one, two and three hours post thawing. To assess the oocyte’s resistance to micro-injection in Group 1, a secondary outcome was the survival rate after “empty” micro-injection (without spermatozoa) performed 3 hours after thawing, specified as the number of intact oocytes after the micro-injection gesture.

We considered the benchmark value of 85% for oocyte survival after manual vitrification and thawing in infertile patients, as suggested by our lab results and the Alpha Scientists [1]. To test the hypothesis that the survival rate is significantly higher (95%) after semi-automated vitrification than manual vitrification (85%), we estimated necessary to include 75 oocytes (150 oocytes total) per group when taking into account the correlation between donors using a McNemar test in the least favorable situation (two-sided test, alpha = 0.05, beta = 0.2, OR = 3, proportion of discordant pairs = 0.2). Sample size estimation was done using Gpower v 3.1.9.7. Categorical variables were expressed as number of cases and percentage of occurrence, and continuous variables as means and standard deviations. To account for donor-related oocyte correlation, mixed models with random effects on donors were estimated (logistic for binary criteria, linear for quantitative criteria). No other adjustments were made in the models to assess the effect of the freezing method. Analyses were performed in R v4.0.3 and using the lme4 package. A *P* value of <0.05 was considered statistically significant.

### RNA isolation and quantification

The sc-RNAseq method was performed as recently described by this team [33]. Briefly, the free-zona pellucida oocyte (after using acidic Tyrode’s solution) was individually placed in a lysis buffer containing MgCl_2_ (4,379,878, Applied Biosystems), DTT, Nonidet P-40 (11,332,473,001, Roche), SUPERase-In (AM2694, Ambion) and RNase-inhibitor (AM2682, Ambion). Then, we performed a reverse transcription reaction (SuperScript III reverse transcriptase - 18,080-044, Invitrogen) and a poly(A) tailing to the 3′ end of the first-strand cDNA (by using terminal deoxynucleotidyl transferase - 10,533-073, Invitrogen). After the second-strand cDNA synthesis, 20 cycles of PCR were performed to amplify the oocyte cDNA using the TaKaRa ExTaq HS (TAKRR006B, Takara) and IS PCR primer (IDT). Following purification with Zymoclean Gel DNA Recovery Kit (ZD4008, Takara), product size distribution and quantity were assessed on a Bioanalyzer using an Agilent 2100 high-sensitivity DNA assay kit (5067–4626, Agilent Technologies). The library preparation (KAPA Hyper Plus Library prep kit) and the sequencing of the scRNA-seq was performed by the ICGex - NGS platform (Curie Institute) on NovaSeq 6000 Illumina sequencer (Illumina, San Diego, USA) for 100-bp paired-end sequencing.

### Bioinformatic analysis

We carried out sequencing quality checks with FastQC [3], and the trimming of adapters and low-quality sequences was done using TrimGalore! [24]. Paired-end reads were aligned onto Human reference genome (hg38) with STAR (v2.5.2) [17] reporting randomly one position, allowing 6% of mismatches. Repeat annotation was downloaded from RepeatMasker and merged with gene annotation from Gencode v29 [19]. The combined file was used as input for quantification with *featureCounts* [26], as recommended in Teissandier et al. [43] [43]. The Transcript Integrity Number (TIN) was estimated to assess post-sequencing RNA quality for each canonical transcript with the RSeQC package [45]. For each sample, we calculated the median non-zero TIN (medTIN) and compared mean medTIN between oocyte groups via mixed linear models with a random effect on donor. The proportion of mitochondrial DNA (%mtDNA) was calculated by dividing the total number of read counts that mapped to the 38 annotated mitochondrial genes with the library size for each sample. Next, only genes with a minimum of 10 reads in at least 3 samples were retained for further analysis. Differential expression analysis was performed after relative log expression normalization of read counts using DESeq2 following the recommendations for paired samples [27]. Log2 fold changes were shrunk with the *lfcShrink* function using the *ashr* method [40] in order to remove the noise associated with fold changes from genes with low expression. Genes were declared as differentially expressed if FDR < 5%. We used *rlog* read counts transformation from DESeq2 as input to perform hierarchical clustering of samples and principal component analysis (PCA). Gene Ontology over-representation analysis was conducted using the *enrichGO* function from the clusterProfiler R package [49]. Protein-protein interaction of differentially expressed genes (DEGs) was mapped using STRING database online tool (version 11.5) [42], selecting a high-confidence minimum required interaction score (≥0.7).

## Supplementary Information

**Additional file 1.** Oocyte surfaces before and after vitrification process.

**Additional file 2.** A. Comparison of mean medTIN between FH and FZ oocytes. Significance was assessed using a linear mixed model with random effects. B. Gene body scRNA-seq read coverage across all samples. C. Comparison of the proportion of mitochondrial DNA (%mtDNA) between FH and FZ oocytes. Significance was assessed using a linear mixed model with random effects.

**Additional file 3 **Semi-automated *vs.* manual vitrification differential expression analysis results for all genes.

**Additional file 4 **Semi-automated *vs.* manual vitrification differential expression analysis results for all genes only for *in vivo* mature oocytes.

**Additional file 5 **FH *vs*. FZ oocytes differential expression analysis results for all genes.

**Additional file 6.** Expression of DNA methylation-related genes across fresh, manual and semi-automated vitrification groups. None of these genes were significantly differentially expressed in the comparisons between semi-automated vs manual and FH vs FZ.

## Data Availability

The datasets generated during the current study are available in the NCBI Gene Expression Omnibus repository (GEO; https://www.ncbi.nlm.nih.gov/geo/) under accession number GSE205417.
