# Supplementary material for: Comparison of oocyte vitrification using a semi-automated or a manual closed system in human siblings: survival and transcriptomic analyses
Source: J Ovarian Res. 2022 Dec 5;15:128. doi: 10.1186/s13048-022-01064-3 (PMC9720994; doi:10.1186/s13048-022-01064-3)
Supplement: Supplementary file 1 — Additional file 1. Oocyte surfaces before and after vitrification process. [file 13048_2022_1064_MOESM1_ESM.docx]

| **Oocyte surfaces (µm^2^)** | **Manual** | **GAVI** | **β (95%CI)** | ***p*-value^a^** |
| --- | --- | --- | --- | --- |
| Before vitrification | 10053.91 (± 757.87) | 9987.90 (± 612.05) | 66.01 (-102.14 ; 234.16) | 0.443 |
| T0 post thawing | 10236.12 (± 781.85) | 10251.20 (± 713.44) | -8.56 (-210.11 ; 192.99) | 0.934 |
| T1 hour | 10157.81 (± 701.70) | 10085.63 (± 688.46) | 77.01 (-106.77 ; 260.79) | 0.414 |
| T2 hours | 10134.40 (± 681.80) | 10065.49 (± 687.80) | 72.16 (-106.44 ; 250.76) | 0.431 |
| T3 hours | 10055.58 (± 656.25) | 10015.83 (± 642.80) | 42.34 (-130.15 ; 214.83) | 0.632 |

For continuous variables, mean (± SD) are presented. 95%CI: 95% Confidence Interval.

^a^ Linear mixed models with random effect on donor were estimated
