# Supplementary figures and images for: Comparison of oocyte vitrification using a semi-automated or a manual closed system in human siblings: survival and transcriptomic analyses
Source: J Ovarian Res. 2022 Dec 5;15:128. doi: 10.1186/s13048-022-01064-3 (PMC9720994; doi:10.1186/s13048-022-01064-3)

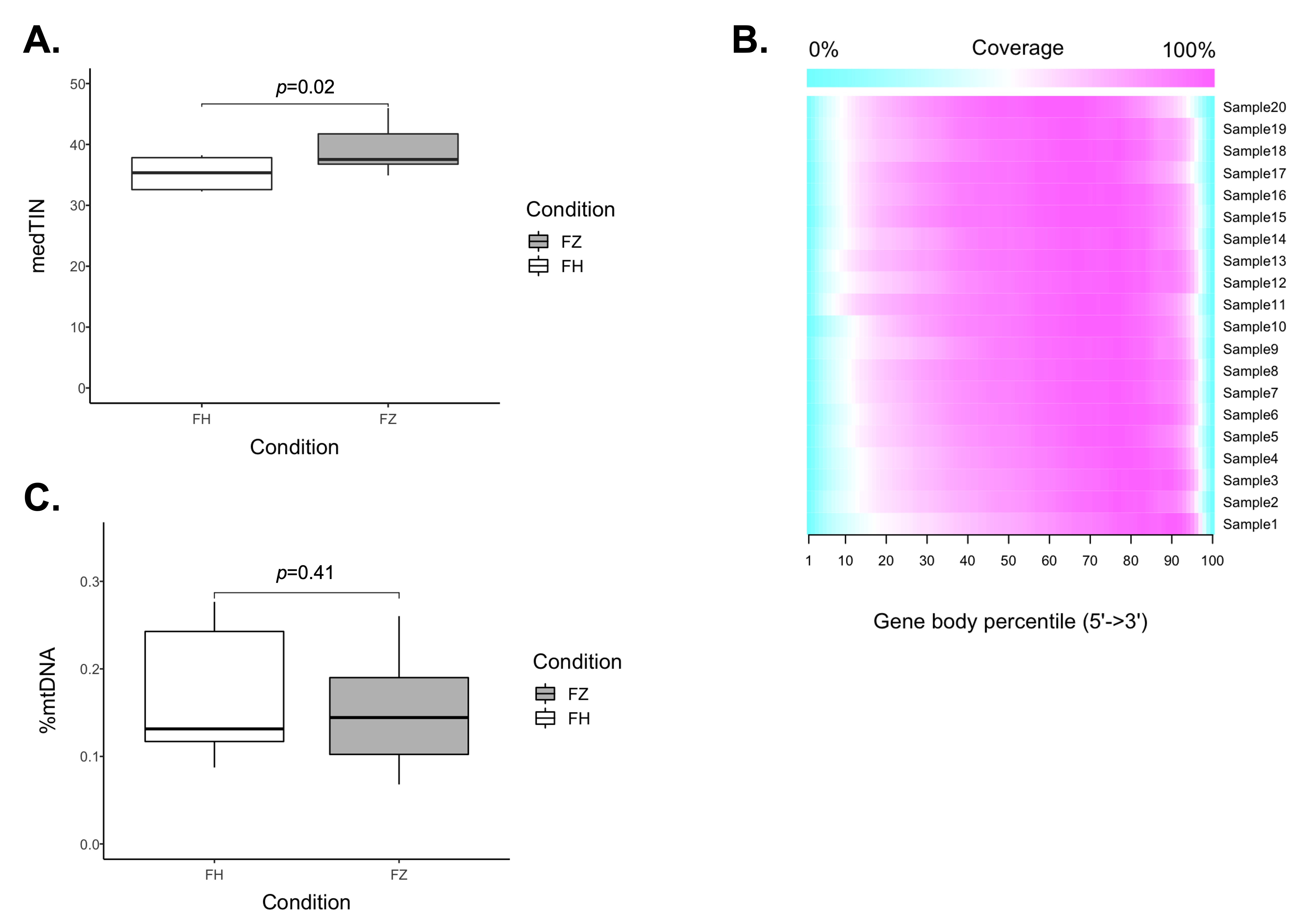

Supplement: Supplementary file 2 — Additional file 2. A. Comparison of mean medTIN between FH and FZ oocytes. Significance was assessed using a linear mixed model with random effects. B. Gene body scRNA-seq read coverage across all samples. C. Comparison of the proportion of mitochondrial DNA (%mtDNA) between FH and FZ oocytes. Significance was assessed using a linear mixed model with random effects. [file 13048_2022_1064_MOESM2_ESM.zip › 1-Additional file 2.jpg]

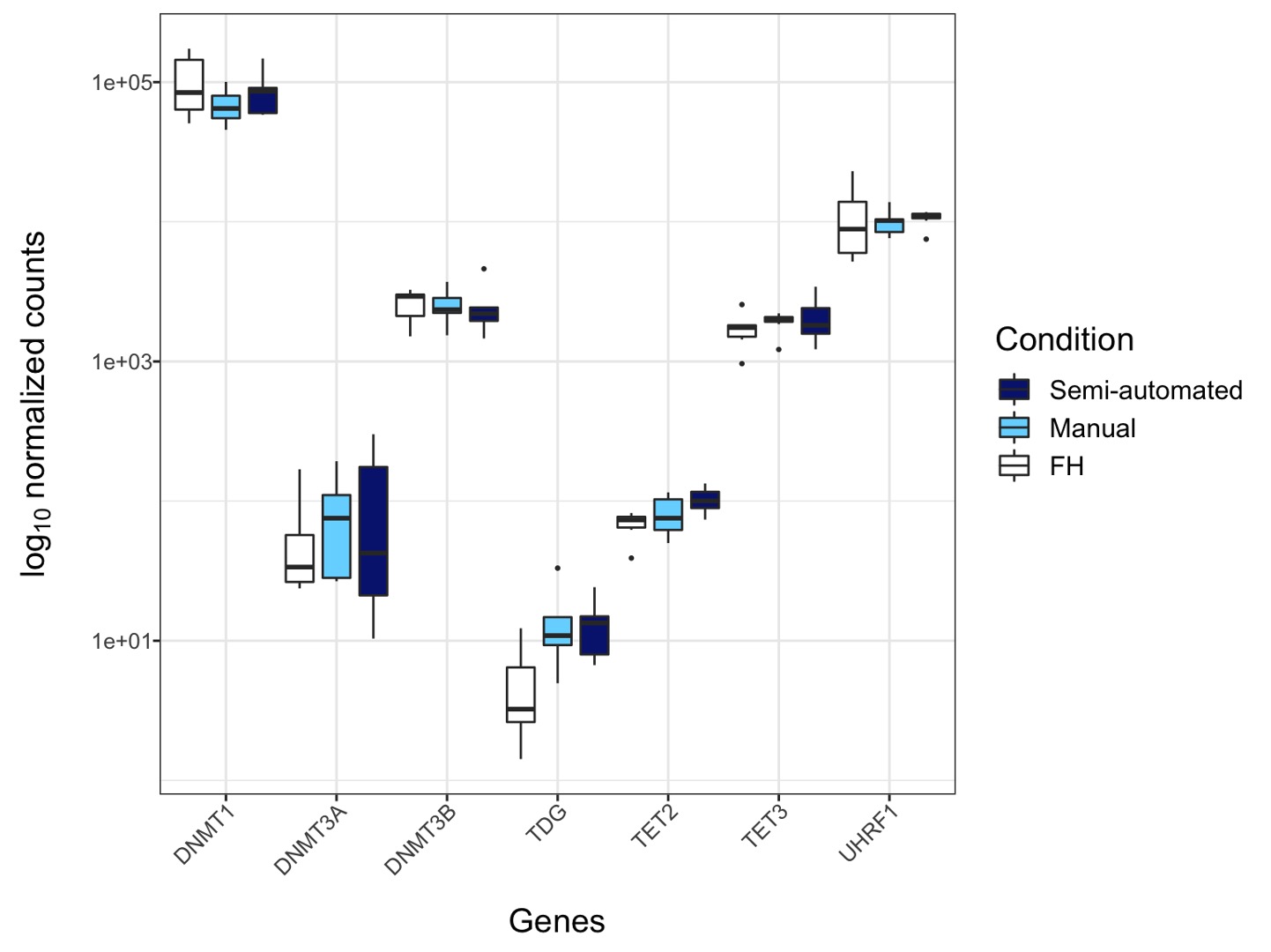

Supplement: Supplementary file 6 — Additional file 6. Expression of DNA methylation-related genes across fresh, manual and semi-automated vitrification groups. None of these genes were significantly differentially expressed in the comparisons between semi-automated vs manual and FH vs FZ. [file 13048_2022_1064_MOESM6_ESM.jpg]
